# Editorial: Biomarkers of Oxidative Stress

**DOI:** 10.3389/fphys.2020.00338

**Published:** 2020-04-22

**Authors:** Magnus Gram, Bo Åkerström

**Affiliations:** ^1^Lund University, Department of Clinical Sciences Lund, Pediatrics, Lund, Sweden; ^2^Section for Infection Medicine, Department of Clinical Sciences, Lund University, Lund, Sweden

**Keywords:** reactive oxygen species, pathological mechanisms, mitochondria, hemoglobin-/heme scavengers, endoplasmatic reticulum, metabolic dysfunction

## Introduction

The term “oxidative stress” is often used to describe physiological conditions where there is an imbalance between oxidants and antioxidants. This can lead to a disruption of redox signaling and induction of molecular damage in cells and tissues (Halliwell and Gutteridge, 2015). Important groups of oxidant molecules are reactive oxygen species (ROS) and free radicals, molecules that are highly reactive due to the presence of unpaired electron(s). The human body is constantly exposed to ROS, free radicals and other oxidants, both exogenously via the environment (food, air, smoke, irradiation, etc.) and endogenously as by-products of normal metabolism. Oxidative stress is involved in many pathological processes, such as inflammation, ischemia/reperfusion and infection, and may lead to development of several pathologic conditions, including neurodegenerative disease, cancer, renal disease, diabetes, cardiovascular diseases and inflammatory bowel disease (Olsson et al., 2012).

The scope of the present Research Topic was to reach out to an audience of scientists working in the field of oxidative stress. We encouraged the submission of papers describing biomarkers of oxidative stress and to discuss their possible usefulness in improving our understanding of disease pathogenesis, aiding in the diagnosis of diseases, developing new therapeutic strategies, and monitoring treatment outcome. A collection of original research and review articles with different approaches, ranging from basic science to evaluation of patient material, contributed to this Research Topic highlighting interesting aspects of biomarkers in oxidative stress. In this editorial, we have summarized the contributions under the following headings: new biomarkers, new pathological mechanisms, hemoglobin/heme-scavengers, and mitochondria, endoplasmic reticulum stress, and metabolic dysfunctions.

### Proposing New Biomarkers

As expected from the Research Topic title, several of the contributions have proposed new biomarkers of oxidative stress. Prasad et al. adapted electron spin resonance spectroscopy and two-dimensional ultra-weak photon emission to detect UV light-induced triplet excited carbonyls and singlet oxygen formation in skin biopsies. This may be a new tool to investigate the effect of UV-induced oxidative stress of human skin. Larsson et al. suggest that levels of the human antioxidant α_1_-microglobulin (A1M) in synovial fluid may be used as a biomarker to predict long-term risk of development of arthrosis after knee injury. Sharma et al. suggest that transthyretin may be a new biomarker with prognostic value for a wide range of oxidative stress-related medical conditions, including Alzheimer's disease and Parkinson. Kalapotharakos et al., finally, show that there are increased plasma levels of the heme-scavenger A1M in high-risk pregnancies and therefore plasma A1M could be a potential biomarker of pregnancy-related diseases.

### Proposing New Pathological Mechanisms

Preeclampsia, a disease of pregnancy which is associated with oxidative stress, was the focus of two contributions each investigating alternative and independent pathological mechanisms. Sánchez-Aranguren et al. could show that soluble Fms-like tyrosine kinase-1 (sFlt-1, also known as vascular endothelial growth factor receptor-1, VEGFR-1), which is elevated in plasma from preeclamptic pregnant women, may contribute to metabolic disturbance and to the development of the disease by triggering mitochondrial ROS-formation in endothelial cells and trophoblasts. Kalapotharakos et al., on the other hand, studied the involvement of extracellular fetal hemoglobin (HbF) originating from placental hemolysis, in the development of preeclampsia. Proposals of new interesting pathological mechanisms of action of the plasma protein transthyretin, commonly known as a transporter of thyroxin and retinol, are reviewed by Sharma et al. Novel oxidative stress-associated pathological mechanisms, related to the release of heme from hemoglobin (Hb), are proposed for knee arthropathies and atherosclerosis, by Larsson et al. and Gáll et al., respectively. Vrbanovic et al. propose an association between salivary oxidative stress and pain in temporomandibular disorders, and Prasad et al. propose novel pathways in ROS-formation during UV-light exposure of skin. Valacchi et al., finally, introduce the novel concept of “OxInflammation” to describe the long-term cross-talk between pro-oxidants, free radicals and inflammatory mediators during inflammation.

### Hemoglobin/Heme-Scavengers

As described above, the contributions by Kalapotharakos et al., Larsson et al. and Gáll et al. discuss the importance of pathological mechanisms related to extracellular Hb and release of heme. In addition, these contributions hypothesize that, when the increased Hb-levels are unmet by the Hb- and heme-scavenger proteins haptoglobin (Hp), hemopexin (Hpx) and/or A1M, excessive hemolysis in placenta, knee-joints or atherosclerotic plaques generate locally increased levels of ROS by reactions between the heme-chelated iron atom and oxygen. The resulting oxidative stress is thought to contribute to preeclampsia (Kalapotharakos et al.), arthrosis of the knee (Larsson et al.) and atherosclerosis (Gáll et al.). The contribution by Maamoun et al. describe the cytoprotective role of the inducible heme-degrading enzyme heme oxygenase-1 (HO-1) as an antioxidant and regulator of ROS-induced toxicity, thereby protecting against endoplasmic reticulum (ER)-stress in diabetic endothelial cells.

### Mitochondria, Endoplasmic Reticulum Stress, and Metabolic Dysfunctions

Chen et al. review the growing knowledge of mitochondrial involvement in modulating oxidative stress and the innate immune response in cardiovascular diseases, autoimmunity, and metabolic syndrome. Mitochondria constitute a “convergent signaling hub” that regulates the cellular redox balance and homeostasis, they argue, and highlight that mitochondria are centrally involved in the driving pathogenic responses upon injury or damage to cells and tissues. As outlined above, Sánchez-Aranguren et al. give an example of this, showing that sFlt-1 targets mitochondria of placental endothelial cells and trophoblasts, disturbs the mitochondrial redox balance, and as a result contributes to the development of preeclampsia. In addition to the mitochondria, two papers in this Research Topic have focused on the ER and the dysfunctional state of this organelle, ER-stress. Maamoun et al. review current knowledge on how the interplay between oxidative stress and ER-stress contributes to aberrant angiogenesis and endothelial dysfunction in diabetes. Gáll et al. show that ROS-induced ER-stress is involved in development of atherosclerosis.

## Conclusion

The above referenced articles show that the Research Topic presents an exciting scientific arena, attracting interest from different aspects and at different levels, from basic to translational research. We hope these articles can contribute to the development of new ideas and advancements in the field.

## Author Contributions

MG and BÅ wrote, revised, and approved the manuscript.

## Conflict of Interest

The authors declare that the research was conducted in the absence of any commercial or financial relationships that could be construed as a potential conflict of interest.
